# Efficacy of intravenous lidocaine in prevention of post extubation laryngospasm in children undergoing cleft palate surgeries

**DOI:** 10.4103/0019-5049.63654

**Published:** 2010

**Authors:** CS Sanikop, Sonal Bhat

**Affiliations:** 1 Department of Anaesthesiology, J.N. Medical College and K.L.E.S.H. and M.R.C, Belgaum - 590 010, Karnataka, India; 2 J.N. Medical College and K.L.E.S.H and MRC, Belgaum - 590 010, Karnataka, India

**Keywords:** Cleft palate surgeries, intravenous lidocaine, laryngospasm

## Abstract

A one-year randomized placebo-controlled trial was conducted to study the effectiveness of intravenous lidocaine in the prevention of post extubation laryngospasm in children, following cleft palate surgeries. Children of age three months to six years were randomly assigned into two groups. Group P placebo (saline) and Group L (Lidocaine), 1.5 mg/kg. A sample size of 74 with n = 37 in each group was selected. The anaesthetic procedure was standardized. At the end of the procedure, three minutes after reversal, the study drug, that is, intravenous lidocaine (1.5 mg/kg) or placebo (saline) was administered and two minutes later the child was extubated. Following extubation for 10 minutes, the haemodynamic parameters, that is, pulse, blood pressure, oxygen saturation, severity of coughing, and laryngospasm were noted. The total reduction of laryngospasm and coughing was 29.9% and 18.92% with IV lidocaine. Significant alterations in haemodynamics and oxygen saturation were noted for 10 minutes, following extubation. Hence, intravenous lidocaine 1.5 mg/kg was effective in the prevention of post extubation laryngospasm in children undergoing cleft palate surgeries.

## INTRODUCTION

Laryngospasm is a serious complication which may be seen following extubation of children under a light plane of anaesthesia.[1] Laryngospasm is a frequently encountered complication in children undergoing upper airway surgery, which, if left untreated, can lead to an increase in morbidity and mortality. Laryngospasm can occur during induction, intubation and extubation. The reported incidence of laryngospasm in patients aged zero to nine years is 17.4%, and is even higher in children between one to three months age.[2] The incidence of laryngospasm after adenoidectomy and tonsillectomy is reported to be as frequent as 21–26%.[3] Children are more prone to airway obstruction, as they have a narrow laryngeal and tracheal lumen that may be blocked by mucosal edema following trauma.[4]

The precipitating factors for laryngospasm are airway manipulation (e.g., intubation or extubation of the trachea), foreign material in the larynx (e.g., blood, secretions) or with light planes of anaesthesia in a non-intubated patient (e.g., regurgitation / vomiting, surgical stimulation, irritated volatile agent, or failure of anaesthetic delivery system).[5]

IV lidocaine being easily available in the operation theater, with an additional advantage of blunting pressor response during laryngoscopy and endotracheal intubation, that is, increase in heart rate and blood pressure, can thus be safely employed in clinical anaesthesia. Studies on the prevention of post extubation laryngospasm with intravenous lidocaine, in paediatric patients undergoing adenoidectomy and tonsillectomy, have been carried out.[6] Information is lacking on the effectiveness of intravenous lidocaine in the prevention of post extubation laryngospasm in children undergoing cleft lip and palate surgery. Hence there is a need for the study.

## METHODS

After obtaining the approval from the hospital ethical committee and the written informed consent from the patients, the study was conducted at the K.L.E.S Hospital and M.R.C. Belgaum.

A sample size of 74 children, aged between three months and six years, with cleft palate of ASA grade one and two were studied. Two groups (n = 37) with the power of 80% and an α value of 0.05 and effect size = 20% reduction was assumed. Patients with untreated upper respiratory tract infections, two or more attempts at intubation and patients requiring post-operative elective ventilation were excluded from the study.

The children were randomly divided into two groups using computer-generated randomization.

Group L – Lidocaine (1.5 mg/kg)

Group P – Placebo (Normal saline)

Noninvasive monitors such as pulse oximeter, electrocardiogram, automated noninvasive blood pressure and end tidal CO_2_ were used throughout the procedure.

The children were pre-oxygenated with 100% O_2_ for three minutes. They were then pre-medicated with Inj. glycopyrrolate 0.005 mg/kg and Inj. ketamine 5 mg/kg intramuscularly 15 minutes prior to securing the intravenous line, with an appropriate-sized cannula. The children were induced with Inj. ketamine 1 mg/kg and Inj. suxamethonium 1 mg/kg. The oral intubation was done with an appropriate-sized R.A.E. tube. The maintenance was carried out with O_2_, N_2_O, Inj. vecuronium 0.1 mg/kg, and I.P.P.V. The neuromuscular blockade was antagonised with Inj. glycopyrrolate 0.01 mg/kg and Inj. neostigmine 0.05 mg/kg.

**Figure 1:**
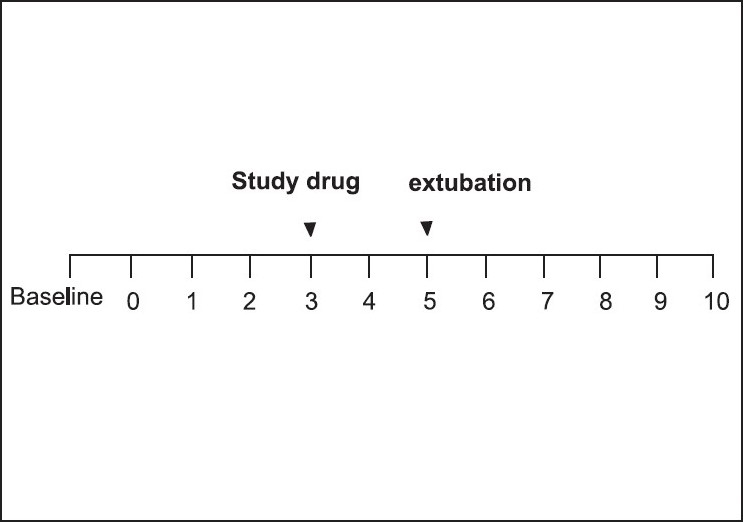
Time interval for administration of reversal (0), study drug (3), and extubation (5), in minutes

Three minutes after the reversal, the study drug (Lidocaine 1.5 mg/kg or normal saline) was administered and the children were extubated two minutes later [Figure 1].

After extubation, 100% oxygen was administered for three minutes.

The following was noted for 10 minutes following extubation.

Haemodynamic vitals, SpO_2_, colour of the child, breathing pattern and activity of the child.

- Coughing was evaluated using:
  The modified four point scale[7] (Tsui Ban C.H., *et al*.)
  0 ⇒ None
  1 ⇒ Slight
  2 ⇒ Moderate
  3 ⇒ Severe
- Laryngospasm was graded using:-
  The four point scale[8] (Tsui Ban C.H., *et al*.)
  0 ⇒ No laryngospasm
  1 ⇒ Stridor during inspiration
  2 ⇒ Total occlusion of cords
  3 ⇒ Cyanosis

### Oxygen desaturation

Oxygen saturation was measured by a Pulse Oximeter. The values of oxygen saturation were regarded as reliable if the pulse waveform was regular. Oxygen saturation (SpO_2_) of < 95% for 30 or more seconds was taken as a desaturation event.[9]

### Statistical analysis

Analysis was done using the student unpaired 't' test, test of proportion and Chi square test.

Demographic data and haemodynamic changes were evaluated using the student unpaired t-test.

Laryngospasm and coughing were evaluated using the test of proportion.

Oxygen saturation was evaluated by the Chi square test.

*P* value < 0.05 was considered statistically significant.

A confidence interval of 95% was used to evaluate the incidence of coughing and laryngospasm.

## RESULTS

There was no significant difference between the groups with respect to age, sex, weight, duration of surgery, anaesthesia [Table 1].

**Table 1:** Results

Demographic data	Group P	Group L	*P* value
Age (Years)	20.05	19.67	0.93
Sex (M:F)	19:18	18:19	M = F
Weight (Kgs.)	8.20	7.47	0.21
Duration of surgery (min.)	90.3	92.8	0.67
Duration of anaesthesia (min.)	104.3	106	0.77
Extubation time (sec.)	139	144	0.66

There was no significant difference between both the groups with respect to age, sex, weight, duration of surgery, anaesthesia and extubation time.

The heart rate and systolic and diastolic blood pressures were well-maintained following extubation in Group L.

The incidence of laryngospasm in Group P was 24.32% and in Group L was 5.71%. Reduction in the incidence of laryngospasm was 18.92% on administration of intravenous lidocaine *P* value = 0.0031 was statistically significant [Table 2].

**Table 2:** Incidence of laryngospasm

		Group P	Group L	Reduction of incidence by
Grade 1	I	10.80%	5.40%	5.40%
	C	(4)	(2)	
Grade 2	I	10.80%	0%	10.80%
	C	(4)	(0)	
Grade 3	I	2.70%	0%	2.70%
	C	(1)	(0)	
Total	I	24.32%	5.40%	18.92%
	C	(9)	(2)	(*P* value = 0.011) (CI = 2 to 35%)

Total reduction in the incidence of laryngospasm was 18.92% and thus considered statistically significant. I = Incidence, C = Number of cases

The incidence of coughing was 40.5% in Group P and 11.43% in Group L. The incidence of coughing was reduced by 28.6% on administration of lidocaine. *P* value = 0.0108 was statistically significant [Table 3].

**Table 3:** Incidence of coughing

		Group P	Group L	Reduction of incidence by
Grade 1	I	24.32%	2.70%	21.62%
	C	(9)	(1)	
Grade 2	I	10.80%	5.40%	5.40%
	C	(4)	(2)	
Grade 3	I	2.70%	2.70%	0%
	C	(1)	(1)	
Total	I	40.54%	10.80%	29.74%
	C	(14)	(4)	(*P* value = 0.0017) (CI = 10.7 to 48%)

I = Incidence, C = Number of cases

Total reduction in the incidence of coughing was 29.74% and thus considered statistically significant.

There was no significant difference in the heart rate preoperatively. There was a significant increase in the heart rate postoperatively at one, two, three, five and ten minutes, following extubation in Group P compared to Group L. Hence, there was a significant difference in heart rate at one, two, three, five and ten minutes following extubation between the two groups [Table 4].

**Table 4:** Heart rate changes

	Group P		Group L		*P* value	
	Mean	S.D.	Mean	S.D.		
Pre op	114.5676	22.25926	115.86	24.802	0.8135	NS
1 min	141.1081	22.86286	127.78	29.099	0.0318	S
2 min	146	20.71768	125.62	27.245	0.00054	S
3 min	139.8378	23.32799	124.05	26.543	0.0082	S
5 min	133.7568	20.38982	122.54	26.8117	0.01465	S
10 min	137.6216	25.02371	119.946	27.4357	0.0164	S

There was a significant difference in the heart rate at two minutes following extubation in both groups. On administration of intravenous lidocaine 1.5 mg/kg, the heart rate was maintained well.

**Figure 2:**
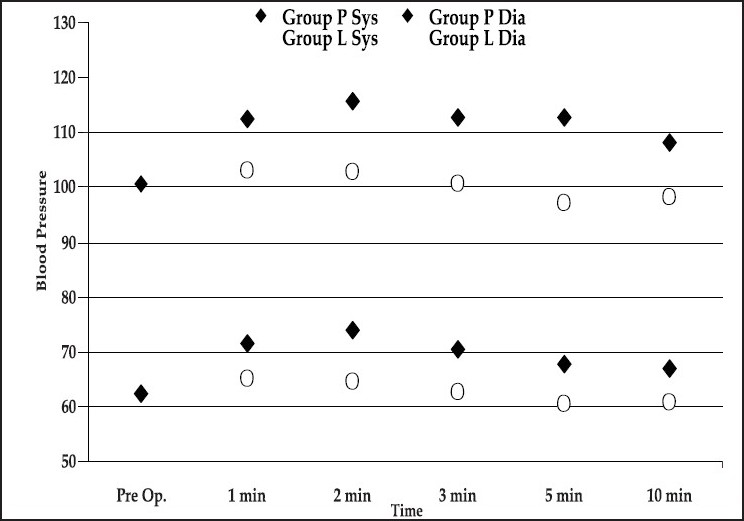
Post extubation blood pressure changes

There was some significant difference in systolic and diastolic blood pressure at one, two, three, five and ten minutes following extubation between the two groups, Group P and Group L [Figure 2].

There was a significant difference in blood pressure at two minutes following extubation in both groups.

**Figure 3:**
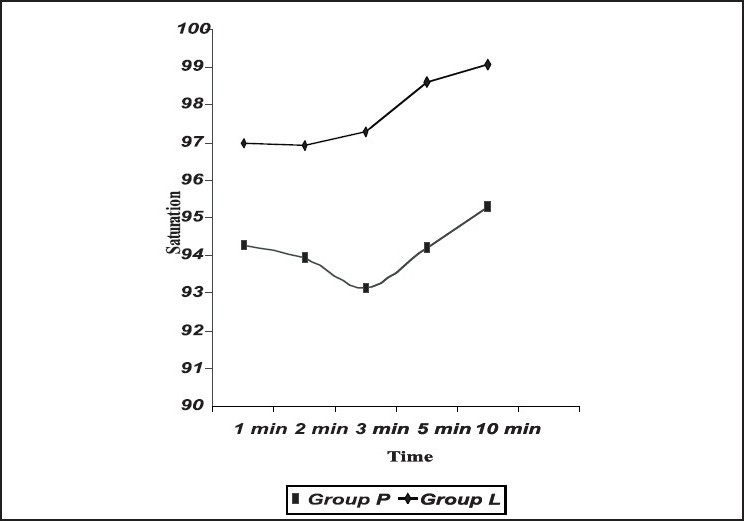
Post extubation oxygen saturation

There was a significant fall in oxygen saturation at the end of three minutes following extubation in Group P. Oxygen saturation was well-maintained throughout after extubation on administration of intravenous lidocaine 1.5 mg/kg [Figure 3].

There was a fall in the oxygen saturation at three minutes following extubation in the placebo group (saline). Oxygen saturation was well-maintained throughout, after extubation, on administration of intravenous lidocaine 1.5 mg/kg.

## DISCUSSION

**Figure 4:**
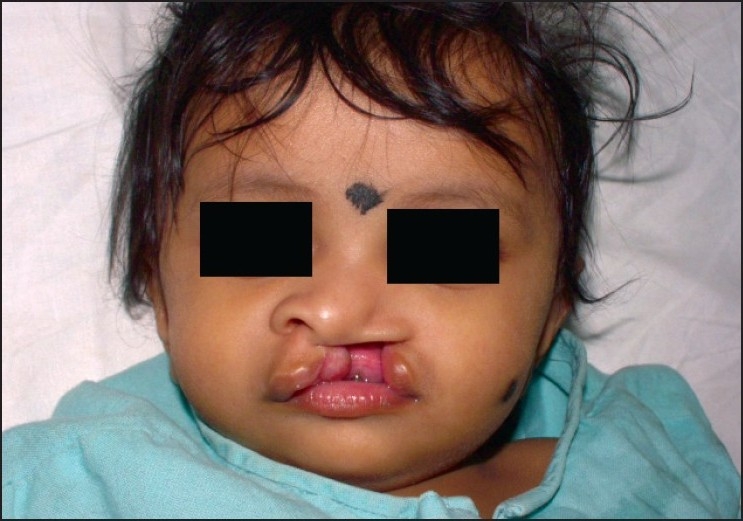
Before the surgery

**Figure 5:**
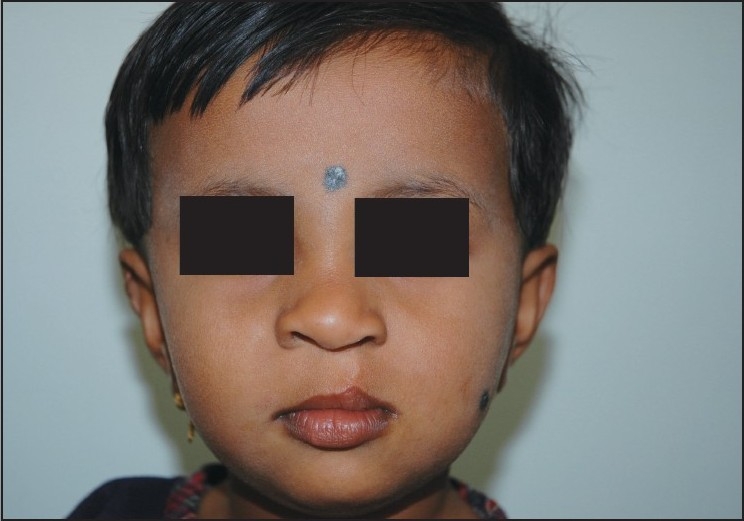
After the surgery

Airway management is one of the most important skills in the field of anaesthesiology. Paediatric airway management remains the most daunting task before the anaesthesiologist [Figures 4 and  5].

Baraka Anis[10] conducted a study on a group of 40 children undergoing tonsillectomy where 20 children were injected with 2% lidocaine bolus one minute prior to extubation and there was no incidence of laryngospasm in the study group, the other twenty were extubated without lidocaine. The incidence of laryngospasm in the control group was found to be 20%.[1]

In our study the incidence of laryngospasm in Group P was 29.32% and in Group L 5.4%. The reduction in incidence was by 18.92%. A *P* value of 0.011 was considered statistically significant. Hence the results were consistent with our study.

Abou Madi MN *et al*.[11] demonstrated that intravenous lidocaine 1 mg/kg administered two minutes before endotracheal intubation prevented coughing and increased blood pressure and heart rate, during and after extubation. In our study there was a significant increase in heart rate, systolic and diastolic blood pressure at one, two, three, five and ten minutes following extubation, in Group P. Paediatric patients with cleft palate usually have upper respiratory tract infections (URTI) and thus have hyper-reactive airways that precipitate laryngospasm.

Laryngospasm occasionally presents atypically and may be precipitated by factors which are not immediately recognised, thereby increasing the potential for patient harm and further complications such as pulmonary aspiration and post-obstructive pulmonary oedema. Hence, the anaesthesiologist needs to take a proactive approach to prevent and terminate laryngospasm.

However awake patients can also experience laryngospasm. Deep extubation provides protection by abolishing coughing and expiratory reflex, but the apnoeic and laryngeal closure reflexes are retained.

A previous study demonstrated that prevalence of active URTI doubles the incidence of laryngospasm.[12]

Plasma concentration of lidocaine at three minutes was 6 – 7.5 microgram/ml and at five minutes, 2 – 3 microgram/ml. Lidocaine 2 mg/kg produced significant depression of the cough reflex and was most effective at 3 – 5 minutes after injection and at seven minutes, and thereafter there was no depression of the cough reflex. The intravenous route was used as it was safer, simple, convenient and most effective.[13]

Hypoxemia frequently occurs after termination of general anaesthesia during the immediate post-operative period.[4]

Tsui Ban CH *et al*.[14] concluded that there was no incidence of coughing, oxygen desaturation, or laryngospasm in children undergoing adenoidectomy and tonsillectomy with a 'No touch' extubation technique. In our study there was a significant fall in oxygen saturation at three minutes following extubation in Group P.

Post-operative coughing may increase arterial pressure, heart rate, intraocular and intra-cranial pressure. Persistent coughing might lead to complications such as laryngospasm.[13]

Ates *et al*.[15] concluded that 5% of the patients had laryngospasm, 22% had coughing and desaturation in children undergoing ophthalmic surgery with a history of frequent upper respiratory tract infection.

In our study the incidence of coughing in Group P was 40.59% and in Group L it was 10.80%. On administration of intravenous lidocaine there was a reduction in the incidence of coughing by 29.74%.

## CONCLUSION

Intravenous lidocaine (1.5 mg/kg) is effective in prevention of post-extubation laryngospasm in children undergoing cleft palate surgeries, with no haemodynamic or SpO_2_ alterations.
